# Assessment of unrecognized myocardial infarction using cardiac magnetic resonance imaging in patients with endstage renal disease

**DOI:** 10.1590/0100-3984.2024.0090

**Published:** 2025-02-27

**Authors:** Ihsan Yuce, Mustafa Keles, Mecit Kantarci

**Affiliations:** 1 Department of Radiology, Ozel Saglik Hospital, Izmir, Turkey; 2 Department of Nephrology, MMT American Hospital, Mersin, Turkey; 3 Department of Radiology, Medical Faculty, Ataturk University, Erzurum, Turkey

**Keywords:** Magnetic resonance imaging/methods, Kidney failure, chronic, Contrast media/metabolism, Myocardial infarction/diagnosis, Ressonância magnética/métodos, Falência renal crônica, Meios de contraste/metabolismo, Infarto do miocárdio/diagnóstico

## Abstract

**Objective:**

To assess the frequency of unrecognized myocardial infarction and identify additional ischemic conditions, as well as to evaluate the efficacy of cardiac magnetic resonance imaging (CMRI) in risk groups, comparing the imaging findings with electro-cardiographic (ECG) and laboratory data in patients with stage 5 chronic kidney disease, also known as end-stage renal disease.

**Materials and Methods:**

This was a prospective single-center study involving 20 patients who were referred to our radiology department to undergo CMRI between June 2010 and December 2011. Resting left ventricular functions and (early and late) myocardial contrast enhancement were assessed in all patients. Laboratory tests and ECG were conducted on all individuals. The mean duration of clinical follow-up was 18 á 4 months.

**Results:**

Pathological results were seen in six (30%) of the patients in our study sample. Scar tissue was identified as a high-risk factor in three patients (15%), and myocardial hibernation was shown to pose a moderate risk in three patients (15%). In the remaining 14 cases, no pathology was identified, and the risk was therefore categorized as low. A statistically significant disparity in mortality rates was observed between the high- and low-risk groups (*p* < 0.05). There were no statistically significant differences between the two groups in terms of the ECG and cardiac biomarkers.

**Conclusion:**

Our findings indicate that CMRI is effective in accurately categorizing risk groups and detecting ischemic conditions, even when such events are not evident on ECG or laboratory tests.

## Abstract (pt)

**Objetivo:**

Nosso estudo teve como objetivo avaliar a frequência de infarto indefinido do miocárdio e identificar condições isquêmicas adicionais, avaliar a eficácia da ressonância magnética cardíaca (RMC) em grupos de risco e comparar os achados de imagem com eletrocardiograma (ECG) e dados laboratoriais em pacientes com doença renal terminal.

**Materiais e Métodos:**

O presente estudo incluiu 20 pacientes encaminhados ao serviço de radiologia para realização de exames de RMC entre junho de 2010 e dezembro de 2011. As funções em repouso do ventrículo esquerdo e a avaliação do realce miocárdico precoce e tardio pelo contraste foram avaliadas em todos os pacientes. ECG e testes laboratoriais foram realizados em todos os indivíduos. Os pacientes foram clinicamente monitorados por um período médio de 18 á 4 meses.

**Resultados:**

Resultados patológicos foram observados em seis indivíduos (30%) do nosso grupo de pesquisa. Tecido cicatricial foi identificado como fator de alto risco em três pacientes (15%). Além disso, foi demonstrado que a hibernação representa um risco moderado em três pacientes (15%). Nenhuma patologia (de baixo risco) foi descoberta nos restantes 14 casos. Observou-se disparidade estatisticamente significante nas taxas de letalidade entre pacientes de grupos de alto e baixo risco (*p* < 0,05). Nenhum achado estatisticamente significante foi obtido no ECG e nas enzimas cardíacas.

**Conclusão:**

Os resultados da nossa pesquisa indicam que a RMC é eficaz na categorização precisa de grupos de risco e na detecção de condições isquêmicas em pacientes, mesmo quando tais eventos não são demonstrados em ECG e exames laboratoriais.

## INTRODUCTION

Cardiac magnetic resonance imaging (CMRI) was initially utilized only to evaluate cardiac morphology and heart movements. In the last decade, it has also started to be widely used in the evaluation of ischemic heart diseases, including myocardial infarction (MI), and there have been innovations in the technology. It has been used successfully to detect the presence and determine the prevalence of myocardial ischemia, to evaluate myocardial viability, to determine ventricular function, and to visualize luminal narrowing in the coronary arteries.

Unrecognized MI (UMI) is characterized by a myocardial scar in patients with no history of MI. Depending on sex, age, and history of coronary artery disease (CAD), the reported prevalence of UMI ranges from 5% to 40%^(1)^. Population-based studies have revealed that the 10-year mortality rate in patients with UMI is 45–55%^(2^,^3)^, equal to or higher than that reported for patients with recognized MI. Taken together, these findings indicate that UMI is a major clinical problem.

Patients with stage 5 chronic kidney disease, also known as end-stage renal disease (ESRD) constitute a high-risk population for CAD, which significantly increases mortality in such patients. Therefore, it is important to identify coronary diseases in this group of patients prior to kidney transplantation.

Electrocardiography (ECG) determination of myocardial biomarker levels, and radionuclide studies—SPECT and PET—can be utilized for the diagnosis of patients with UMI. However, those methods have some limitations for the identification of infarction, including the low sensitivity of ECG, the fact that blood tests are positive only for the first few days after the event, the low resolution of SPECT, and the high dose of ionized radiation used during PET. In addition to being an operator-dependent examination, ECG does not provide information about the epicardial arteries or microvascular circulation. When only conventional tests are used for preoperative risk stratification in renal transplantation candidates, MI can be missed^(1)^. Therefore, there is a need for examinations that are more sensitive, are more specific, and do not involve the use of ionizing radiation. Because CMRI is a noninvasive imaging method that does not use ionizing radiation and can show not only scar tissue but also ischemic events including myocardial hibernation and stunning, as well as because of its high spatial resolution, it is a significant imaging method that can be used in the evaluation of cardiac viability in patients with ESRD. Unspecified MI shows the highest prevalence in diabetes mellitus, hypertension, female gender, and the elderly population^(4)^. In our country, the most frequent causes that play a role in the etiology in patients with ESRD are diabetes mellitus, chronic glomerulonephritis, and hypertension. Hence, the incidence of UMI increases in patients with ESRD. CAD is a significant cause of mortality in this group of patients.

Kidney transplantation is the primary treatment method in patients with ESRD. Studies indicate that, in patients who also have CAD, the mortality rate increases following surgery. Therefore, it is crucial to identify high-risk candidates prior to transplantation.

All of this suggests that UMI is a critical clinical problem in patients with ESRD. Therefore, the objective of this study was to determine the frequency of UMI, to compare the ECG findings with the levels of cardiac biomarkers and to contribute to the prognosis by using MRI to evaluate cardiac viability in patients with ESRD scheduled to undergo kidney transplantation.

## MATERIALS AND METHODS

### Study design

This was a prospective single-center study involving 20 patients scheduled to undergo kidney transplantation who were transferred from the nephrology department to the radiology department of our hospital between June 2010 and December 2011 to undergo CMRI. The study was conducted in accordance with the Declaration of Helsinki, and the study protocol was approved by the local ethics committee (Reference no. 2010-02/03). Written informed consent was obtained from each participant.

The inclusion criteria were being ≥ 18 years of age and having been referred by a nephrologist for pre-transplantation evaluation. Patients with a history of ischemic or infective heart disease (e.g., endocarditis and myocarditis) were excluded. Of the 20 patients evaluated, 12 (60%) were women, with a mean age of 44.5 years, and eight (40%) were men, with a mean age of 52.6 years. The causes of renal failure included diabetes mellitus, in eight patients (40%); hypertension, in five (25%); nephrolithiasis, in four (20%); and chronic glomerulonephritis, in three (15%).

The main objective of the CMRI protocol was to assess cardiac viability and function. On the same day, cardiac biomarker levels were determined and ECG was performed. Dialysis commenced on the day after the CMRI scan.

### CMRI protocol and image analysis

Left ventricular functions, as well as myocardial first-pass and late contrast enhancement were assessed in all patients at rest in a 1.5-T MRI scanner (Magnetom Avanto; Siemens Healthineers, Erlangen, Germany). Long- and short-axis left ventricular images were acquired at rest with gradient echo and steady-state free precession sequences. The parameters were as follows: repetition time/echo time, 3.8/1.6 ms; flip angle, 45°; receiver bandwidth, 125 kHz; slice thickness, 8.0 mm; and interslice gap, 2.0 mm. Gadoterate meglumine (0.1 mmol/kg, Dotarem; Guerbet, Roissy, France) was injected through the antecubital vein, after which early (0th min) and late (10th min) short axis images were obtained. The images obtained were transferred to a workstation (Syngo; Siemens Healthineers), and cardiac viability and left ventricular functions were evaluated with the help of the software Argus (Siemens Healthineers). No artificial intelligence-assisted technologies were employed in our study.

### ECG

All patients underwent standard 12-lead ECG. Cardiovascular Health Study criteria were utilized in order to identify MI^(5)^. Accordingly, we looked for ST-T segment changes, as well as for major Q wave or minor Q wave abnormalities. Other ECG parameters were not evaluated in our study. After CMRI, blood samples were collected from all of the patients to determine the levels of routine cardiac biomarkers (troponin I, troponin T, creatine kinase, creatine kinase-myocardial band, and myoglobin).

### Classification of patients according to CMRI findings

On the basis of the CMRI findings, the patients were divided into three groups, by risk: low, moderate, and high. The low-risk group included patients without a pathology identified on CMRI; the moderate-risk group included patients with perfusion defects at rest, including stunning and hibernation; and the high-risk group included patients with scar tissue. Stunned myocardium is defined as prolonged postischemic dysfunction after reperfusion, whereas hibernating myocardium is defined as a chronically ischemic and depressed myocardium related to narrowing of the coronary artery. Although there is myocardial dysfunction in both entities, perfusion is normal in stunned myocardium (reperfusion after occlusion) and impaired in hibernating myocardium.

### Clinical follow-up

Our patients were followed clinically in order to identify newly developing ischemic events and mortality. The patients who underwent transplantation during the study period were distinguished from those who remained on the transplant waiting list. The mean duration of clinical follow-up was 18 á 4 months.

### Evaluation of all acquired findings

All of the data acquired were assessed by a board of radiologists and cardiologists who are specialists in their field. Cardiac ischemia (stunning or hibernation), subendocardial-transmural scar, if any, additional pathologies (valve dysfunction, mass lesion, pericardial effusion, etc.) were investigated in the patients with ESRD, and a plan was made to collect data regarding their frequency in those patients. In addition, the high-risk transplantation candidates were identified and the risk groups were compared with in terms of the cardiac biomarker and ECG data.

### Statistical analysis

The chi-square test was employed to compare the risk groups and mortality rates on the basis of the CMRI findings. There was insufficient numerical data for a statistical evaluation between the CMRI findings and the ECG-troponin values. All statistical analyses were performed with the MedCalc software version 12.2.1.0 (MedCalc, Ostend, Belgium). The level of significance was set at *p* = 0.05.

## RESULTS

**Figure 1:**
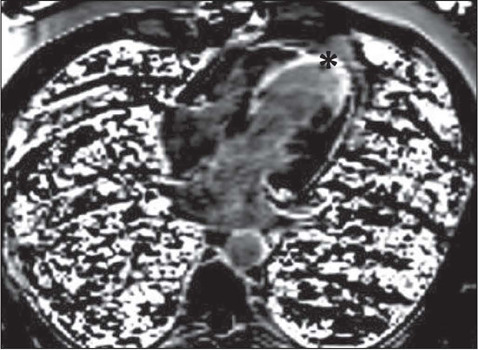
In the four-chamber images taken at minute 10 in a 55-year-old male patient, hyperintense areas consistent with full-thickness scar tissue are observed in the left ventricular apex and interventricular septum (asterisk).

**Figure 2:**
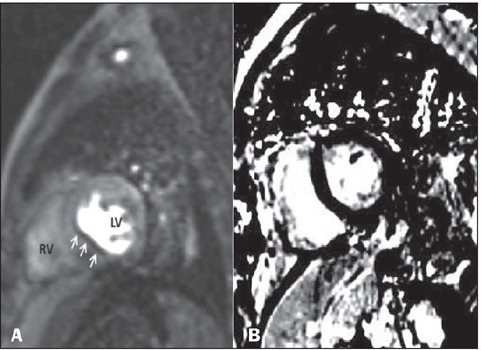
A: A defect in myocardial perfusion (arrows) is observed in the 0–3-minute early contrast short-axis images of a 72-year-old male patient. A lack of contrast enhancement is observed in the late images in B as well. In the patient with dyskinesia in the same region on cine images, the appearance was deemed to favor myocardial hibernation. (RV, right ventricle; LV, left ventricle).

Pathological findings were obtained in six (30%) of the 20 patients evaluated. On CMRI, scar tissue (Figure 1), considered a high-risk factor, was detected in three (15%) of the patients; hibernation (Figure 2), considered a moderate-risk factor, was observed in three (15%); and no pathology (low risk) was noted in CMRI in the remaining 14 patients (70%).

Table 1 shows the demographic, clinical, and imaging data for all of the patients in the sample. One patient (5%) tested positive for troponin and had a pathological Q wave on ECG. Detailed information of left ventricular functions are shown in Table 2.

**Table 1:** Detailed clinical, imaging, and follow-up findings in patients with ESRD.

Patient no.	Risk group	Age (years)	Sex	ECG findings	CMRI findings	Cardiac biomarkers	Clinical outcome	Transplant
1	Moderate	57	Male	Normal	Hibernation	Normal	Survival	No
2	Low	39	Female	Normal	Normal	Normal	Survival	No
3	Low	33	Female	Normal	Normal	Normal	Survival	No
4	Moderate	72	Male	Normal	Hibernation	Normal	Survival	No
5	Moderate	55	Female	Normal	Hibernation	Normal	Survival	No
6	High	57	Female	Normal	Scar tissue	Normal	Survival	No
7	Low	30	Female	Normal	Normal	Normal	Survival	No
8	Low	57	Female	Normal	Normal	Normal	Survival	No
9	Low	47	Female	Normal	Normal	Normal	Survival	No
10	High	55	Male	Pathologic Q wave	Scar tissue	Troponin-positive	Death	Yes
11	Low	64	Male	Normal	Normal	Normal	Survival	No
12	Low	56	Female	Normal	Normal	Normal	Survival	No
13	Low	38	Male	Normal	Normal	Normal	Survival	No
14	High	39	Female	Normal	Scar tissue	Normal	Death	No
15	Low	61	Male	Normal	Normal	Normal	Survival	No
16	Low	29	Male	Normal	Normal	Normal	Survival	Yes
17	Low	18	Female	Normal	Normal	Normal	Survival	Yes
18	Low	49	Female	Normal	Normal	Normal	Survival	No
19	Low	55	Female	Normal	Normal	Normal	Survival	No
20	Low	45	Male	Normal	Normal	Normal	Survival	No

**Table 2:** Detailed information of mean left ventricular functions.

Parameter	Mean á standard deviation
Left ventricular mass index (g/m^2^)	92.2 á 7.1
End-systolic volume index (mL/m^2^)	43.4 á 10.6
End-diastolic volume index (mL/m^2^)	92.5 á 29.7
Ejection fraction (%)	57.3á 9.3
Stroke volume index (mL)	45.5 á7.2

The patients in our sample were followed for an average of 18 á 4 months. Kidney transplantation was performed in three patients (15%). One of those patients was in the high-risk group and died from cardiac causes 20 months after transplantation. In addition, one patient who did not undergo transplantation and was also in the high-risk group died during the study period.

The mortality rate was significantly higher among the patients in the high-risk group than among those in the low-risk group (*p* = 0.012); two of the three patients in the high-risk group died during the study period, whereas there were no deaths in the low-risk group. There was no statistically significant difference between the moderate-risk group and the high-risk group.

## DISCUSSION

In most cases, UMI is detected by ECG in patients who have no clinical history of MI. The reported prevalence of UMI ranges from 5% to 40%^(1)^. Advanced age, diabetes mellitus, and hypertension are risk factors for the condition. Population-based studies have shown that the 10-year mortality rate is 45–55% among patients with UMI, equal to or higher than that reported for patients with recognized MI^(2^,^3)^. These data indicate that UMI is a major clinical entity.

Because patients with ESRD are at high cardiovascular risk, cardiovascular disease is one of the leading causes of death after transplantation in such patients. In comparison with ECG, conventional echocardiography, and nuclear scintigraphic techniques, CMRI is more sensitive for the detection of UMI^(6^,^7^,^8)^, with very high sensitivity even for the detection of small infarcts. Ibrahim et al.^(9)^ found that the sensitivity of CMRI was greater than that of single-photon emission computed tomography (SPECT) for the detection of small infarctions and non-Q-wave infarctions (85% vs. 46%). The overall sensitivity of CMRI in that study was 97%. In an international multicenter study, Kim et al.^(10)^ found that the sensitivity of CMRI for the detection of chronic MI was 94%. Therefore, in recent studies, CMRI has been the clinical gold-standard method for the diagnosis of UMI^(11)^.

In transplant candidates, UMI can be missed if only conventional tests (ECG, SPECT, echocardiography, etc.) are utilized in the preoperative risk assessment. Therefore, we aimed to detect UMI by using CMRI in patients with ESRD.

The disadvantages of ECG are related to non-Q wave MI and its low sensitivity in showing pathology after a certain period following ischemia. Those of SPECT include its low spatial resolution, its inability to show small infarcts, significant radiation exposure to patients, and attenuations caused by the diaphragm. In addition, echocardiography has low sensitivity for the detection of chronic MI. For UMI screening, coronary angiography is not recommended if patients do not have cardiac symptoms^(12)^. None of the patients in our sample presented with cardiac symptoms.

Technological developments in the last decade have, in part, led to CMRI is a that has becoming increasingly popular as a noninvasive imaging method for the diagnosis of cardiovascular diseases. Its popularity has also increased because it can, in a single examination, provide all of the same data obtained with all other noninvasive imaging methods together. In addition to the fact that it does not involve the use of ionizing radiation, CMRI can provide satisfactory images even without the administration of contrast media. Its other advantages are that it has higher spatial, temporal, and soft-tissue resolution than do other imaging methods.

The CMRI technique utilized to detect MI is known as delayed enhancement. In late contrast enhancement, breath hold sequences are typically implemented. The images consist of approximately 10 short-axis images acquired during myocardial contractions and additional long-axis images. The typical dose of contrast agent is 0.1–0.2 mmol/kg^(13^,^14)^. The assessment of late contrast enhancement is basically based on the fact that hyperintense infarct areas, unlike normal myocardium, are observed in the images obtained 10–15 min after intravenous gadolinium injection, due to impaired cellular integrity and increased extracellular area. Late contrast enhancement can be observed in ischemic and nonischemic myocardial diseases. In ischemic diseases, the infarct area spreads from the subendocardium to the subepicardium and in areas that match the coronary artery irrigation area. In nonischemic diseases, isolated midmyocardial or epicardial involvement can be observed in areas that do not match the irrigation area of the coronary arteries.

The presence of microvascular obstruction areas after MI enhances the incidence of recurrent MI, congestive heart failure, stroke, and death. Microvascular obstruction seems to be characterized by hypointense areas within hyperintense scar tissue on late contrast images. These areas, which cannot be identified with SPECT, significantly affect the prognosis^(13^,^14)^.

Transmurality of the infarct can also be detected with CMRI. Studies have shown that the ratio between the infarct area and the overall myocardial thickness has an inverse relationship with functional myocardial recovery. For instance, it has been reported that functional recovery occurred in 60% of patients with ≤ 25% infarction, in 42% of those with 25–50% infarction, in 10% of those with 50–75% infarction, and in only 1.7% of those with ≥ 75% infarction^(15)^.

The CMRI findings were positive for MI in six (30%) of the 20 patients in our study sample. Myocardial scar tissue was detected in three patients (15%). Our results are consistent with data in the literature^(1)^. One of the three patients with myocardial scar tissue had a transmural infarction, and the other two had nontransmural infarctions. Other than the CMRI evidence, no positive findings were found in the nontransmural infarctions. It is quite challenging to detect small infarct areas in ESRD patients with other viability-determining tests. There are numerous studies comparing CMRI with SPECT and PET in this context, and those studies have demonstrated that CMRI is more sensitive for detecting small infarcts^(16^,^17^,^18)^.

In our study, pathological Q and troponin values were significantly positive for MI on ECG in one patient (5%), in whom CMRI also revealed a scar. Nevertheless, in the other two patients in whom we detected scarring and in the patients in whom hibernation was detected, no significant findings were noted in relation to troponin, other cardiac biomarkers, or ECG parameters. In one study of this topic, ECG was found to have a sensitivity of 22.2% and a specificity of 98.1%^(1)^. As can be understood from this ratio, it is inevitable that nontransmural infarctions will be missed on ECG. Laboratory tests and ECG findings are more suited for acute settings, whereas CMRI is superior for chronic ischemia and scar detection^(1^,^16^,^18)^.

Although troponin values are expected to be above normal in patients with ESRD, we believe that this would not be due to reduced renal clearance alone. Therefore, high troponin levels require further cardiac investigation in such patients. Various studies have shown that elevated troponin is directly associated with cardiac mortality and morbidity^(19)^. In the present study, a kidney transplant recipient with elevated troponin levels died after approximately 20 months of clinical follow-up. The other death occurred in a patient with myocardial scar tissue who did not undergo kidney transplantation. In a similar study, conducted by Andrade et al., death occurred in 19 (26%) of 72 patients with ESRD during a follow-up period of 4.0–77.9 months^(1)^.

In our risk classification according to CMRI findings, a statistically significant difference was noted between the low-risk group and the high-risk group. Because mortality rates are higher among patients at high risk, preoperative and postoperative cardiac control, as well as precautions prior to transplantation, become even more crucial in such patients. The two patients in our sample who died during the study period (one who had undergone transplantation and one who was on the transplant waiting list) were in the high-risk group. Studies regarding this subject have indicated that scar tissue detected by CMRI is strongly correlated with major cardiac events and mortality^(20)^.

Stunning and hibernating myocardium, including transient left ventricular dysfunction as a consequence of acute and chronic ischemia, were also evaluated in our study. As previously mentioned, hibernated myocardium was identified in three (15%) of the 20 patients in our sample. In a review of the literature, we found no studies describing pre-infarct conditions seen on CMRI in patients with ESRD. Therefore, we believe that ours is the first such study. In cases in which infarct transmurality is low, preoperative evaluation with ECG and cardiac biomarkers can produce erroneous (false-negative) results. The CMRI-based risk classification system we have created could be utilized as a predictor of preoperative and postoperative mortality. With CMRI, information can be obtained not only about infarction but also about ischemic dysfunction.

Recent studies have employed CMRI T1 mapping to investigate myocardial fibrosis in patients with ESRD. Myocardial fibrosis has important prognostic value in such patients^(21^,^22^,^23)^. Such studies will also be beneficial to patients with ESRD who are going through the transplantation process.

In comparison with other tools, the diagnostic utility of CMRI will enhance pre- and post-transplantation management. Medical or invasive treatment (conventional angiography) and routine follow-up will provide enhanced secondary prevention and long-term management after the diagnosis of UMI^(10^,^24^,^25)^, with the objective of having a positive effect on post-transplant survival.

Our study has some limitations. First, the patient population was relatively small and there were a limited number of clinical events, and those factors limited the statistical power of the study, particularly concerning the observed mortality outcomes. Therefore, our results, especially the UMI-related mortality rate, need to be confirmed in large-scale multicenter studies. In addition, the delayed myocardial enhancement technique is limited in ESRD because of a safety issue, given that nephrogenic systemic fibrosis has been associated with gadolinium administration. However, according to the American College of Radiology contrast media guidelines, if a contrast-enhanced MRI examination is to be performed in a patient with ESRD on chronic dialysis, injection of group I agents is contraindicated, and the committee recommends the use of a group II agent^(26^,^27)^. In our study, we used a group II agent (gadoterate meglumine). The risk of nephrogenic systemic fibrosis is extremely low when using a group II agent. The American College of Radiology Committee on Drugs and Contrast Media also recommends that gadolinium-enhanced MRI examinations be performed as closely before a regularly scheduled hemodialysis as is possible, because dialysis can improve gadolinium clearance. We recommended dialysis on the same day after the MRI scan. In addition, the lowest dose of gadolinium should be used in at-risk patients, and it should generally not exceed the recommended single dose^(26)^.

## CONCLUSION

In light of all of this information, we strongly suggest that CMRI be performed for detecting UMI in patients with ESRD. The use of the risk classification applied in our study provides important information to the transplantation team in terms of cardiac monitoring. This study makes an important contribution to the literature, and our findings could help guide clinicians in dealing with the diagnosis of UMI in patients with ESRD. However, there is a need for large-scale multicenter studies comparing CMRI and other modalities in this context.
